# Functional Analysis of Promoter Variants in Genes Involved in Sex Steroid Action, DNA Repair and Cell Cycle Control

**DOI:** 10.3390/genes10030186

**Published:** 2019-02-28

**Authors:** Yosr Hamdi, Martin Leclerc, Martine Dumont, Stéphane Dubois, Martine Tranchant, Guy Reimnitz, Penny Soucy, Pauline Cassart, Manon Ouimet, Daniel Sinnett, M’Hamed Lajmi Lakhal Chaieb, Jacques Simard

**Affiliations:** 1 Department of Molecular Medicine, Faculty of Medicine, Université Laval, Genomics Center, CHU de Quebec-Université Laval Research Center, 2705 Laurier Boulevard, Quebec City, QC G1V 4G2, Canada; yosr.hamdi.82@gmail.com (Y.H.); martine.dumont@crchudequebec.ulaval.ca (M.D.); stephane.dubois@crchudequebec.ulaval.ca (S.D.); martine.tranchant@crchudequebec.ulaval.ca (M.T.); guy.reimnitz@crchul.ulaval.ca (G.R.); guy.reimnitz@crchul.ulaval.ca (P.S.); 2 Department of Mathematics and Statistics, Faculty of Science and Engineering, Université Laval, 1045 Av. de la Médecine, Quebec City, QC G1V 0A6, Canada; martin.leclerc.5@ulaval.ca (M.L.); lajmi.lakhal@mat.ulaval.ca (M.L.L.C.); 3 Division of Hematology-Oncology, Research Center, Sainte-Justine University Health Center, 3175 Chemin de la Côte-Sainte-Catherine, University of Montreal, Montreal, H3T 1C5, Canada; cassart.pauline@gmail.com (P.C.); manon.ouimet@recherche-ste-justine.qc.ca (M.O.); daniel.sinnett@umontreal.ca (D.S.)

**Keywords:** breast cancer, promoter variants, candidate genes, functional analysis, *cis*-regulatory effects

## Abstract

Genetic variants affecting the regulation of gene expression are among the main causes of human diversity. The potential importance of regulatory polymorphisms is underscored by results from Genome Wide Association Studies, which have already implicated such polymorphisms in the susceptibility to complex diseases such as breast cancer. In this study, we re-sequenced the promoter regions of 24 genes involved in pathways related to breast cancer including sex steroid action, DNA repair, and cell cycle control in 60 unrelated Caucasian individuals. We constructed haplotypes and assessed the functional impact of promoter variants using gene reporter assays and electrophoretic mobility shift assays. We identified putative functional variants within the promoter regions of estrogen receptor 1 (*ESR1*), *ESR2*, forkhead box A1 (*FOXA1*), ubiquitin interaction motif containing 1 (*UIMC1*) and cell division cycle 7 (*CDC7*). The functional polymorphism on *CDC7*, rs13447455, influences *CDC7* transcriptional activity in an allele-specific manner and alters DNA–protein complex formation in breast cancer cell lines. Moreover, results from the Breast Cancer Association Consortium show a marginal association between rs13447455 and breast cancer risk (*p* = 9.3 × 10^−5^), thus warranting further investigation. Furthermore, our study has helped provide methodological solutions to some technical difficulties that were encountered with gene reporter assays, particularly regarding inter-clone variability and statistical consistency.

## 1. Introduction

Over the last two decades since the discovery of the first high-risk breast cancer susceptibility genes breast cancer 1 (*BRCA1*) and *BRCA2*, an extensive body of literature has grown regarding the causes of hereditary and familial breast cancer [1]. The discovery of these two genes has provided basic and thorough insight in the pathways of carcinogenesis [2]. One of the major observations that was derived from these studies is the marked variability in the penetrance of these genes among carriers sharing the same mutation, suggesting that breast cancer risk may be modified by multiple genetic and non-genetic factors. Moreover, it is now well-established that the genetic component of breast cancer risk is due to a combination of rare variants conferring high to intermediate risk of breast cancer with more common variants conferring a lower risk. In fact, most of the unresolved fraction of the breast cancer familial relative risk might likely be explained by a polygenic model involving a combination of many common low-risk variants that together, may explain a substantial percentage of breast cancer genetic susceptibility [3]. The known high- and intermediate-risk variants have so far been associated with nearly one-third of the total breast cancer risk [4,5,6,7,8]. Low-risk susceptibility loci recently identified account for ~16% of the overall genetic component of breast cancer [9,10,11,12]. Thus, additional susceptibility variants might still be identified, and the molecular mechanisms accounting for the role of single nucleotide polymorphisms (SNPs) in the risk of cancer are still currently investigated.

Disease-associated genetic variants are likely to induce either qualitative structural and/or functional modifications of proteins and/or quantitative changes in cellular levels. Indeed, expression profiling and genome-wide mapping studies have shown that strong heritable factors govern differences in gene expression levels in mammalian species [13]. Furthermore, many large-scale studies have shown that most of the common breast cancer-associated variants appear to lie within non-coding gene regions [14,15]. Indeed, two regulatory polymorphisms (rSNPs), rs2981578 in the fibroblast growth factor receptor 2 (*FGFR2*) gene and rs554219 in the *CCND1* gene may be causally related to breast cancer risk [16,17]. In both cases, the mechanism involved appears to be the binding of transcription factors that control the expression level of the target gene. Moreover, two recent studies performed by our group led to the identification of two new loci, 4q21 and 11q22.3 that show evidence of association with overall breast cancer risk and with the modification of breast cancer risk in *BRCA1* mutation carriers, respectively. In both studies, the associated variants are non-coding variants associated with differential allelic expression [18,19]. In addition, in vitro studies suggest that a high percentage of rSNPs lie within the core and proximal gene promoter regions, and 90% of the validated functional *cis*-regulatory polymorphisms have been shown to lie within the 2-kb proximal promoter [20]. Moreover, most of the breast cancer-associated loci identified so far include genes involved in sex steroid action [21], DNA repair and cell cycle control [22,23], which are three well-documented pathways involved in hormonal regulation and genomic integrity. Interestingly, many of the proteins belonging to these pathways interact directly or indirectly with the BRCA1 or BRCA2 proteins [24,25].

In attempt to identify additional *cis*-acting rSNPs, we have studied the potential role of variants located within the promoter region of 24 genes critically required for DNA repair and cell cycle, as well as genes involved in estrogen-regulated cell proliferation, e.g., in the control of estrogen bioavailability and action. Since a single variant in one candidate gene might not be solely responsible for the full genetic variability relative to a given phenotype, we characterized the regulatory haplotypes (rHaps) for each studied promoter using gene reporter assays and electrophoretic mobility shift assays (EMSA). 

## 2. Materials and Methods

### 2.1. Identification of Promoter Polymorphisms 

To identify rSNPs, PCR amplification (Long Expanded PCR Kit, Roche Diagnostics, Laval, QC, Canada) was performed for regions upstream from the transcription start site of each gene (up to 2.7 kb) in DNA from a population panel of 60 unrelated individuals of Northern and Western European ancestry (Centre d’Etude du Polymorphisme Humain [CEPH]/Utah families, HAPMAPPT01) [26]. The International HapMap DNAs were purchased from the Coriell Institute for Medical Research (Camden, NJ, USA). 

PCR-amplified products were then sequenced using the ABI Prism 3730xl DNA Analyzer automated sequencer (ThermoFisher Scientific, Markham, ON, Canada) and the BigDye Terminator v3.1 fluorescence-based sequencing method (ThermoFisher Scientific). Sequence data was analyzed using the Staden package. 

### 2.2. Accession Numbers

In this study, we analyzed the promoter regions of the following 24 genes (See Table S1 for the HGVS names and other accession numbers of each gene). *ESR1*: NM_001122741, *ESR2*: NM_001437, *FOXA1*: NM_004496, *PGR*: NM_000926, *AR*: NM_001011645, *UIMC1*: NM_016290, *CDC7*: NM_003503, *NBN*: NM_002485, *RAD51*: NM_002875, *BRCC3*: NM_024332, *ATR*: NM_001184, *PALB2*: NM_024675, *RAD51C*: NM_058216, *MRE11A*: NM_005591, *H2AFX*: NM_002105, *ATM*: NM_000051, *UBE2N*: NM_003348, *BABAM1*: NM_014173, *NELFB*: NM_015456, *BRE*: NM_004899, *BRIP1*: NM_032043, *MDC1*: NM_014641, *RPS6KA2*: NM_001006932, *RNF8*: NM_003958.

### 2.3. Linkage Disequilibrium Analysis and Haplotype Estimation

The *r*^2^ statistics of the Haploview 4.0 software [27] were used for haplotype block identification by calculating the pairwise linkage disequilibrium for each variant sequence pair. The block default algorithm as defined by Gabriel et al was selected [28]. The *Tagger* tag SNP selection algorithm of Haploview was used to select a minimal set of tag SNPs [29]. Haplotype reconstruction and frequency estimation was performed using the Phase 2.1.1 software [30]. This program estimates haplotype frequency using a Bayesian algorithm. For all genotyped individuals, haplotypes were estimated using SNPs with a minor allele frequency (MAF) ≥ 5%. Regulatory haplotype inference was performed using the PHASE v.2.1 software. 

### 2.4. Subcloning and Reporter Plasmid Construction 

Following sequencing and characterization, rHap fragments were subcloned into the pGL3-Basic Firefly Luciferase reporter vector (Promega, Madison, WI, USA). The resulting constructs were verified by sequencing to confirm the presence of the expected haplotypes. After sequencing the resulting constructs, we observed spurious variants that appeared due to mistakes while copying DNA by the Polymerase enzyme. To overcome this problem, we optimized our PCR conditions by using a mix of two polymerases of high fidelity. In brief, PCR amplification of the studied promoters have been performed in a final volume of 50 µL (5 uL of Buffer, 2.5 uL of each dNTP (10 mM), 3.5 uL of each primer (10 uM) 15 uL of Betaine, 5.75 uL H_2_O, 1.25 uL of the Fastart polymerase mixed with 1 uL of Pfu polymerase and finally 5 uL of DNA sample (20 ng/uL) have been added. PCR conditions have been optimized as follow: an initial denaturation at 94 °C for 2 min 30 s, followed by 10 cycles of [10 s at 94 °C, 30 s at the annealing temperature and 3 min at 68 °C], followed by 25 cycles of [15 s at 94 °C, 15 s at 56 °C and 3 min at 68 °C], then one cycle at 68 °C for 7 min.

Constructs were then purified using a Sigma (Sigma-Aldrich, Oakville, ON, Canada) Plasmid Purification kit prior to transfection. For several genes, a number of different clones (up to six) corresponding to each rHAPs were amplified, sequenced and subcloned into independent constructs.

### 2.5. In Silico Prediction of Putative Transcription Factor Binding Sites 

Using the MatInspector software [31] as a transcription factor binding site (TFBS) prediction tool, we searched for potential rSNPs with a predictable impact on putative TFBS through either complete loss of the latter and/or via the gain of a novel TFBS. Altered transcription factor binding elements showing significant predicted scores were selected for further functional analysis.

### 2.6. Cell Culture 

Two human cancer cell lines, MCF7 (ER^+^ breast cancer adenocarcinoma) and Hela (cervix adenocarcinoma) were used for transient transfection experiments. MCF7 cells were grown in DMEM-F12 (1:1) medium (Wisent, St-Bruno, QC, Canada) supplemented with 5% fetal bovine serum (FBS), penicillin/streptomycin (100 IU/mL-1%, *w*/*v*), Hepes and 1 nM estradiol. HeLa cells were grown in Eagle’s Minimum Essential Medium (Wisent, St-Bruno, QC, Canada) enriched with 5% FBS, 1% L-glutamine and penicillin/streptomycin (100 IU/mL−C1%, *w*/*v*). Both cell lines were grown at 37 °CC in a 5% CO_2_ atmosphere and 95% relative humidity.

### 2.7. Transient Transfection and Luciferase Reporter Assays 

MCF7 cells were transfected with Lipofectamine^®^ 2000 according to the manufacturer’s protocol (Invitrogen, Burlington, ON, Canada), whereas HeLa cells were transfected with polyethylenimine (ExGen 500; ThermoFisher Scientific − Fermentas). Cells were plated in 24-well plates (6 × 10^4^ cells/well) and incubated for 24 h to reach 50–70% confluence at the time of transfection. Both cell lines were co-transfected with 1 ug of each haplotype-specific construct and 10 ng of either CMV-driven Renilla luciferase pRL-CMV or pRL-null vectors (Promega) (ratio 100:1) to control for transfection efficiency. The choice of control vector to monitor transfection efficiency was based on optimal expression levels and the absence of cross-talk with the promoter under study. Similar experiments were performed with the empty promoterless pGL3-Basic plasmid (Promega) and the SV40-driven Firefly Luciferase pGL3-control plasmid (Promega, Madison, WI, USA) as negative and positive controls, respectively. Cells were harvested 24 h post-transfection and luciferase reporter gene activity was measured with the Dual-Luciferase Reporter Assay System (Promega) according to the manufacturer’s instructions, using an Infinite^®^ 200 luminometer (Tecan; Mânnedorf, Switzerland). The Renilla luciferase control reporter activity was used as an internal control to normalize results of the firefly luciferase activity. Results are expressed as the ratio of firefly luciferase activity divided by the Renilla activity and are presented as the mean relative luciferase activity from four independent replicates. The promoterless pGL3-Basic vector was used to measure basal (background) expression levels of the gene of interest. Each experiment was performed at least twice using one or more clones for the same rHaps, when available. 

### 2.8. Statistical Analysis

To determine the statistical significance of the variability between rHaps and between independent clones of the same rHap, each combination of clone number and rHap was considered as one category of a single fixed effect called cloneHap. Data were analyzed with a mixed model including the fixed effect of cloneHap and the random effect of experiment number. When the effect of cloneHap was significant, we assessed pairwise luciferase activity differences between rHaps and between different clone numbers corresponding to the same rHap using the post-hoc Bonferroni procedure. Haplotype activity with a *p* < 0.01 relative to reference haplotype (H1) was considered statistically significant (see Supplementary Materials). 

### 2.9. Electrophoretic Mobility Shift Assay 

DNA-protein interactions were studied using crude nuclear extracts from HeLa and/or MCF7 cells incubated with 5’-end 32P-radiolabeled double-stranded oligonucleotides corresponding to the sequences encompassing each SNP site tested, using the Gel Shift Assay System (Promega) according to the manufacturer’s instructions. Nuclear extracts (10 µg) were incubated with 35 fmol of the labeled probe in the binding buffer (50 mM Tris-HCl (pH 7.5), 5 mM MgCl2, 2.5 mM EDTA, 2.5 mM DTT, 250 mM NaCl, 0.25 ug/uL polydeoxyinosinate-polydeoxycytidylate and 20% glycerol) in a total volume of 10 µL for 20 min at room temperature. Prior to incubation with the radiolabeled probe, a 50-fold molar excess of the unlabeled target oligonucleotide probe, corresponding unlabeled mutant probe, or target-irrelevant oligonucleotide probe was added to minimize non-specific radiolabel binding. DNA-protein complexes were resolved on a 6% non-denaturing polyacrylamide gel (acrylamide:bisacrylamide 37.5:1) in 1× Tris-glycine-EDTA buffer (190 V at 4 °C), and analyzed using the Cyclone Plus Storage Phosphor System (Perkin Elmer, Woodbridge, ON, Canada).

### 2.10. Functional Annotation Using Public Databases

Publicly available genomic data was used for functional annotation. The following regulatory features were obtained for breast cell types from ENCODE and NIH Roadmap Epigenomics data through the UCSC Genome Browser: Chromatin Hidden Markov Modelling (ChromHMM) states, DNase I hypersensitivity sites, histone modifications of epigenetic markers more specifically commonly used marks associated with enhancers (H3K4Me1 and H3K27Ac) and promoters (H3K4Me3 and H3K9Ac), and transcription factor chromosome immunoprecipitation-sequencing (ChiP-seq) data. Predicted enhancer-promoter determined interactions were obtained from the Integrated Method for Predicting Enhancer Targets (IM-PET) described in He et al. [32]. Overlaps between SNPs and ChromHMM and IM-PET data were performed using the annotation tool VEXOR [33]. 

## 3. Results

### 3.1. Identification of Regulatory Single Nucleotide Polymorphisms and Haplotype Estimation

Using a panel of 60 unrelated Caucasian individuals from families registered at the Centre d’Etude du Polymorphisme Humain (CEPH)/Utah, we amplified and sequenced a region of ~2.5 kb, upstream of the transcription start site of 24 genes involved in sex steroid action, DNA repair and cell cycle control (Table S2). For each promoter region, several potential rSNPs were identified. Table S1 provides information on the identified SNPs, including a list of the SNPs observed for each gene promoter, their position and minor allele frequency. From these SNPs, rHAPs were estimated for each gene and major rHAPs with frequency >5% were generally used for further functional analysis using gene reporter assays (details on SNP identification and estimation of the rHaps are shown in Table S1). For the mediator of DNA damage checkpoint 1 (*MDC1*) gene, only one major haplotype (95%) was estimated and therefore this gene was excluded from further functional analysis.

### 3.2. Gene Reporter Assays and Inter-Clone Variability 

To assess the functional allelic differences between the various rHaps, the promoter activity for each gene was determined using luciferase reporter assays in two human tumor cell lines, namely MCF7 breast cancer cells and HeLa cervix adenocarcinoma cells. 

Despite nearly two decades of investigation, no consensus has been reached among the research community regarding the actual definition of a functionally significant difference in promoter activity for the expression of a given gene. In the present study, we used the criteria defined by Hoogendoorn et al. [34,35] to assess the promoter activity of the various alleles or genes. This includes a minimum threshold of a 1.5-fold increase in reporter activity compared to the promoterless (negative) control as evidence for promoter activity. Such a fold increase is equivalent to a gene dosage increase from two to three copies. Moreover, Hoogendoorn et al. suggested that a significant fold increase should be met across numerous replicates from independent clones [35]. Indeed, an important source of variability in expression levels was observed between independent clones of the same promoter haplotype despite being determined altogether under the same experimental conditions. In fact, extrinsic factors such as those responsible for variations in cell culture conditions or transfection efficiency can markedly affect the level of expression measured for each independently cloned gene, which stresses the importance of using independent clones in reporter gene expression experiments. 

**Figure 1:**
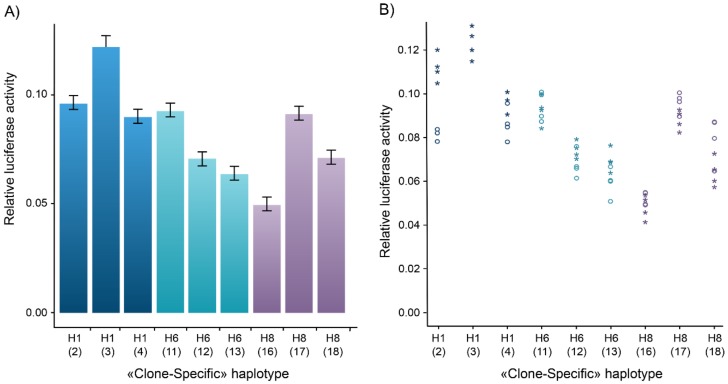
Inter-clone variability observed in transient transfection assays. (**A**) Bars and error bars represent the mean and standard error estimated with the mixed model. (**B**) Stars and circles represent raw data from the two independent experiments respectively. A typical example of the variability in gene expression observed between independent clones of the same haplotype illustrating the relative luciferase activity of clone-specific haplotypes of the *PALB2* gene promoter. Three clones of each haplotype were subcloned and transfected in the MCF7 human breast cancer cell line. The three clones containing the same haplotype sequence should display similar gene expression levels. However, we observed a reproducible difference in the reporter gene expression level between the clones of the same haplotype, and for several genes. Inter-clone variability is observed for the *PALB2* promoter haplotypes H1-3 vs. H1-4, and H8-16 vs. H8-17. To take this unexpected effect into account, we devised and used the statistical method described in this study (see Supplementary Materials).

For instance, results from the gene reporter assays for the partner and localizer of BRCA2 (*PALB2*) gene promoter in MCF7 breast cancer cells are a case in point that illustrates the presence of substantial inter-clone variability between the three independent clones obtained for each of the three *PALB2* rHaps (Figure 1). Except for H1-4 and H1-2, all the other pairwise differences between clones of PALB2-specific haplotypes were significant, with a 1.8-fold difference in the expression levels detected between *PALB2* H8-16 and *PALB2* H8-17. With regard to the above-mentioned criterion, obtaining multiple independent clones of a given promoter region could be particularly challenging due to the limits imposed by low cloning efficiency and plasmid rearrangement. The statistical approach described in the methods section was developed to address this issue when the desired number of replicates cannot be reached in practice (Supplementary Materials). Given the fact that promoter activity may vary considerably depending on the presence of enhancers or silencers and interactions between multiple activator or inhibitor proteins, some promoters may be ubiquitously expressed while others are cell line-specific. The task of extensively characterizing all the transcription factor motifs that can drive transcription in the promoters assayed is daunting. In the current study, we assumed that a quantitatively similar trend in the expression level of the rHaps in both MCF7 and HeLa cell lines might be more suggestive of the presence of an active TFBS.

### 3.3. Transcriptional Activity of Major Regulatory Haplotypes

Reporter gene expression results for all rHaps are presented in Table 1. Data are expressed as the mean activity of each rHap relative to the most frequent haplotype denoted H1. Activity relative to the pGL3-Basic promoterless (negative) control is also shown. As shown in Table 1, genes were classified in three groups according to the presence of a statistically significant differential expression of a given haplotype in both cell lines, in only one cell line or when no significant differential expression was observed in either cell line. 

Overall, these results showed that the promoter region of three genes belonging to the sex steroid hormone action pathway (estrogen receptor 1 (*ESR1*), *ESR2* and forkhead box A1 (*FOXA1*)) and six genes involved in cell cycle progression and in DNA repair (cell division cycle 7 (*CDC7*), serine/threonine kinase (*ATR*), MRE11 homolog, double strand break repair nuclease (*MRE11A*), *PALB2*, RAD51 paralog C (*RAD51C*) and ubiquitin interaction motif containing 1 (*UIMC1*)) displayed at least one regulatory haplotype with significant differential allelic expression in both cell lines. The progesterone receptor gene (*PGR*), as well as the BRISC and BRCA1 A complex member 1 (*BABAM1*), BRCA1/BRCA2-containing complex subunit 3 (*BRCC3*), BRISC and BRCA1 A complex member 2 (*BRE*), negative elongation factor complex member B (*NELFB*), nibrin (*NBN*), RAD51 recombinase (*RAD51*), ring finger protein 8 (*RNF8*) and ribosomal protein S6 kinase A2 (*RPS6KA2*) genes exhibited cell-specific expression in either MCF7 or HeLa cells only (Table 1). 

Despite the particularly high promoter activity observed for the Ataxia Telangiectasia Mutated serine/threonine kinase (*ATM*), BRCA1 interacting protein C-terminal helicase 1(*BRIP1*) and H2A histone family member X (*H2AFX*) genes, no significant variation in rHap expression was observed for these genes nor for ubiquitin conjugating enzyme E2 N (*UBE2N*) compared to the expression of their corresponding H1 haplotype. Moreover, the promoter activity of the androgen receptor gene (*AR*) was below the significance threshold of 1.5-fold increase in reporter activity compared to the promoterless control that was set as evidence of activity. For this reason, no additional experiments were performed for this gene. 

Extensive analyses, including in silico analysis and EMSA assays, were performed on the promoters showing significant differential allelic expression in both cell lines. The results of these analyses, summarized in Table 2, are detailed in the following section.

### 3.4. Sex Steroid Hormone Action Genes

Four haplotypes were identified for the *ESR1* promoter, eight haplotypes for the *ESR2* promoter, and six for the *FOXA1* promoter.

**Figure 2:**
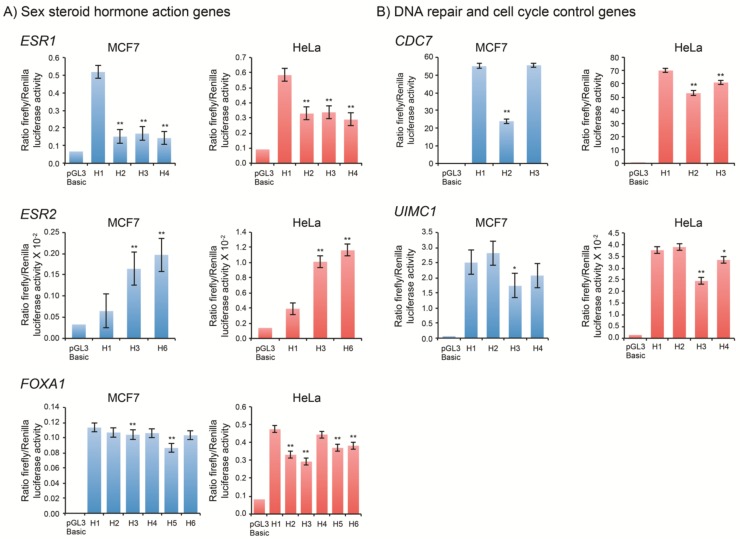
Gene promoter haplotype activity assessed by gene reporter assays. (**A**) sex steroid action, (**B**) DNA repair and cell cycle control genes. Relative luciferase activity of promoter haplotypes was measured following transient transfection in MCF7 and HeLa cells. The empty promoterless pGL3-Basic vector was used as negative control. Results are expressed as relative luciferase activity, i.e., the ratio of firefly/*Renilla* luciferase activity. Bars and error bars represent the mean and standard error from either independent experiments and /or several independent clones in at least one experiment, respectively. The haplotype analyzed shows significant promoter activity compared to the wild-type haplotype H1 according to a mixed model analysis described in material and methods (* *p* < 0.01; ** *p* < 0.001) *ESR1*: Estrogen receptor 1; *FOXA1*: Forkhead box A1; *CDC7*: Cell division cycle 7; *UIMC1*: Ubiquitin interaction motif containing 1.

**Figure 3:**
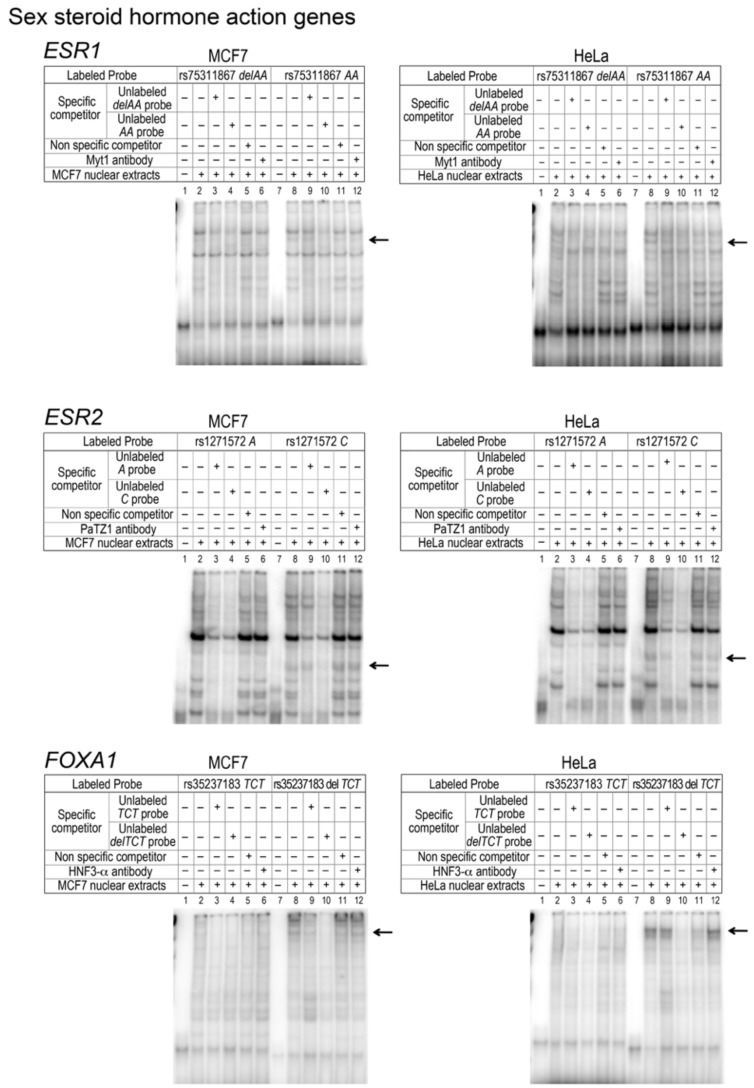
Representative EMSA analysis showing DNA–protein interactions in the promoter region of the selected genes involved in sex steroid hormone action. *ESR1*, *ESR2* and *FOXA1* as genes related to sex steroid action pathway. Labeled double-stranded oligonucleotide probes for each of the respective variants tested were incubated with nuclear extracts from MCF7 and HeLa cells. The unlabeled probes used to determine non-specific radioisotope binding (at a 50-fold molar excess) are indicated for each lane. Specific competitors corresponding to the unlabeled allele-specific probes and a non-specific double-stranded oligonucleotide competitor were used for each experiment. Fast-migrating unbound probes are found at the bottom of the gel whereas protein–DNA complexes formed display a slower mobility. Black arrows indicate probe-specific differential protein binding. The Protein Atlas (https://www.proteinatlas.org) and ProteomicsDB (https://www.proteomicsdb.org) databases report protein expression data for hepatocyte nuclear factor 3 (HNF3) in HeLa cells, and PATZ1 in both HeLa and MCF7.

Luciferase assays of the three *ESR1* rHap, namely H2, H3 and H4, showed a significant decrease (~70%) in their transcriptional activity compared to H1 (Figure 2A). These three haplotypes carry the same variation, namely insAA-rs75311867. In silico analysis using MatInspector as a TFBS prediction tool showed that the “AA-deletion” results in loss of MYT1 transcription factor binding site. EMSA analysis was then performed and provided evidence for a decrease in relative mobility induced by the presence of the rs75311867, as shown in Figure 3. However, supershift assays using an MYT1 antibody failed to show any decrease in the electrophoretic mobility of protein-DNA complex bands. 

In the case of the *ESR2* promoter, the three most common haplotypes (H1, H3 and H6) represented 92% of the observed haplotypes. H3 differs from H1 at the V4-rs1271572 position, whereas H6 differs from H1 at the V4-rs1271572 and V5-rs66615803 positions. Gene reporter analysis led to assign a significant increase of promoter activity to haplotypes carrying the minor allele of rs1271572 (H3 and H6) (Figure 2A). EMSA experiments showed differential protein binding at rs1271572 but not rs66615803 (Figure 3). TFBS analysis indicated that rs1271572 might alter the recognition motifs of the POZ/BTB And AT Hook Containing Zinc Finger 1 (PATZ1) transcription factor. Supershift assays using a PATZ1 antibody failed to detect the decrease in the electrophoretic mobility pattern in EMSA expected for the binding of PATZ1 to rs1271572. Therefore, the mechanism by which rs1271572 alters *ESR2* promoter activity may be due to either the binding of other transcription factors or via a different transcription regulatory mechanism.

Six different haplotypes were identified for the *FOXA1* promoter. Gene reporter assays showed that H3 and H5 were associated with a decrease of the *FOXA1* expression level in both MCF7 and in HeLa cells (Figure 2A). Both rHaps are characterized by an AGA insertion (rs35237183) as well as the presence of the V6-rs10145379 variant. To identify the *cis*-acting element responsible for the observed changes in *FOXA1* promoter activity, we assessed whether rs35237183 could modify protein binding in MCF7 and HeLa cells, using EMSA. These assays showed differential binding of the minor allele in both cell lines. rs35237183 was predicted to alter the binding site of a set of transcription factors among which the most interesting was the hepatocyte nuclear factor HNF3-α (i.e., the protein product of *FOXA1*). Supershift analysis using a HNF3-α antibody failed to show that rs35237183 affected the binding between the *FOXA1* promoter and HNF3-α transcription factor (Figure 3). Additional functional analysis should be undertaken to identify the specific transcription factor that may bind to this sequence variant.

### 3.5. DNA Repair and Cell Cycle Control Genes

Among the 19 studied genes that were involved in either DNA repair or cell cycle control, six (*CDC7*, *UIMC1*, *ATR*, *MRE11A*, *PALB2*, *RAD51C*) displayed at least one regulatory haplotype with significant differential allelic expression in both MCF-7 and HeLa cell lines. 

**Figure 4:**
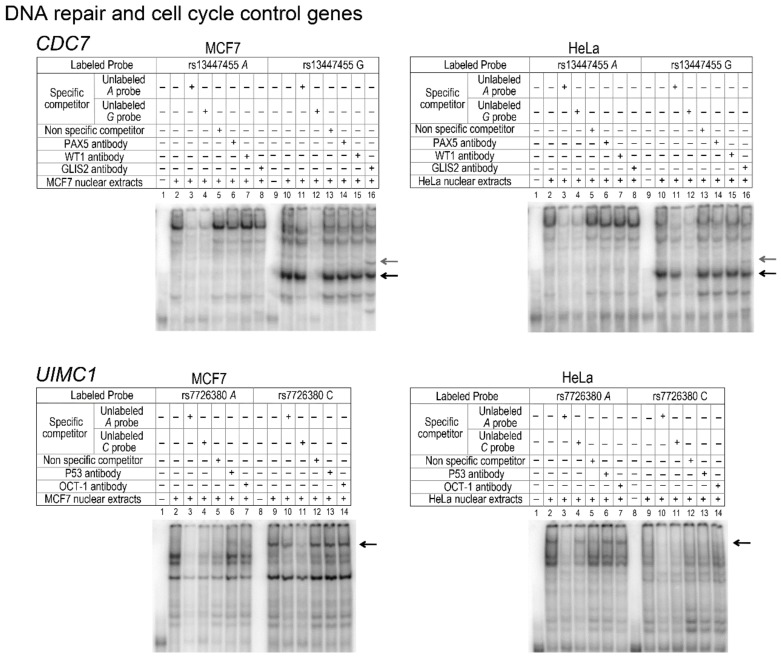
Representative EMSA analysis showing DNA–protein interactions in the promoter region of the selected genes involved in DNA repair and cell cycle control. *CDC7* and *UIMC* as genes involved in DNA repair and cell cycle control pathways. Labeled double-stranded oligonucleotide probes for each of the respective variants tested were incubated with nuclear extracts from MCF7 and HeLa cells. The unlabeled probes used to determine non-specific radioisotope binding (at a 50-fold molar excess) are indicated for each lane. Specific competitors corresponding to the unlabeled allele-specific probes and a non-specific double-stranded oligonucleotide competitor were used for each experiment. Fast-migrating unbound probes are found at the bottom of the gel whereas protein–DNA complexes formed display a slower mobility. Black arrows indicate probe-specific differential protein binding. Bands containing antibody-protein-DNA complexes are highlighted by grey arrows. The Protein Atlas (https://www.proteinatlas.org) and ProteomicsDB (https://www.proteomicsdb.org) databases report protein expression data for GLIS2, Oct-1 in HeLa cells, and p53, Oct-1 in MCF7. No expression data was available for Paired Box 5 (PAX5) or Wilms Tumor 1(WT1) transcription factors in either cell lines.

**Figure 5:**
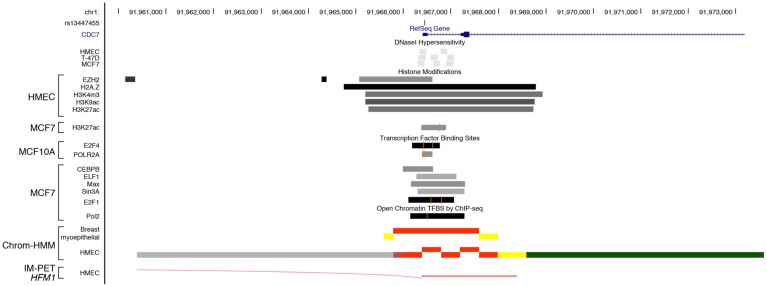
Functional annotation of SNP rs13447455. Functional annotation was performed using data from the Encyclopedia of DNA Elements (ENCODE) and NIH Roadmap Epigenomics projects. From top to bottom, epigenetic signals overlapping rs13447455 included DNase clusters in HMEC, T47D and MCF7 cells; histone modifications in HMEC, MCF10A and MCF7 cell lines; overlap between rs13447455 and transcription factor binding sites in MCF10A and MCF7 cells; chromatin state segmentation by Hidden Markov Model (ChromHMM) in breast myoepithelial and HMEC cells, where red represents an active promoter region, yellow a poised enhancer and green a transcribed region respectively (the detailed color scheme of chromatin states is described in the UCSC browser). Predicted enhancer-promoter determined interactions in HMEC cells, as defined by the integrated method for predicting enhancer targets (IM-PET) are shown.

Three different haplotypes were estimated for *CDC7*. The H1 haplotype differs significantly from H2 at V7-rs13447450 and V10-rs13447455, two variants that are in perfect linkage disequilibrium (*r*^2^ = 1) (Figure 2B). Further functional analysis showed that rs13447450 did not impact transcriptional activity of the *CDC7* promoter (data not shown), whereas rs13447455 displayed an intriguing functional effect. Indeed, EMSA showed a clear gel shift associated with its minor “G” allele (Figure 4). Using TFBS prediction tools, we identified GLIS Family Zinc Finger 2 (GLIS2) as a putative transcription factor whose binding could be disrupted by the presence of rs13447455. Supershift assays using a GLIS2 antibody showed that the latter indeed interacted in a specific manner with the rs13447455-G allele. Thus, binding of GLIS2 to the *CDC7* promoter region studied herein may potentially impact *CDC7* gene expression. Paired Box 5 (PAX5) or Wilms Tumor 1(WT1) transcription factors were also predicted to be disrupted by the variant rs13447455; however, supershift assays did not provide any evidence of binding between antibodies to either of the two candidate transcription factors and the variant. It should be noted however that no data was available for the expression of these proteins in HeLa and MCF7 cells and thus we cannot refute the possibility that the absence of binding is due to the lack of expression of these proteins in these cell lines. Further functional annotation using VEXOR, a web application for the prioritization of functional variants (http://romix.genome.ulaval.ca/vexor), has shown that rs13447455 overlaps with several regulatory features in breast cell lines (Figure 5). Indeed, this variant overlaps with DNase hypersensitivity sites in HMEC, T47D and MCF7 cells, as well as with several histone modification marks, including modifications associated with actively transcribed promoters (H3K4me3 and H3K9Ac) in human mammary epithelial cells (HMEC), and enhancers (H3K27Ac) in HMEC and MCF7 cells. Analysis of Encyclopedia of DNA Elements (ENCODE) data shows that rs13447455 was located within ChIP-seq peaks for binding of transcription factors E2F4 and POLR2A in MCF10A cells, and E2F1, POLR2A, CEBP, ELF1, MAX and SIN3A in MCF7 cells. However, the variant did not directly lie in the DNA binding motifs identified for these transcription factors within these regions. Moreover, ChromHMM data also predict that this variant lies within an active promoter region in breast cell lines in HMEC and breast myoepithelial cell lines. Predicted enhancer-promoter interactions overlapping with rs13447455 were observed in HMEC cells with the promoter of ATP Dependent DNA Helicase Homolog (*HFM1*), a gene located approximately 240 kb upstream *CDC7*. Taken together, these data, along with the functional results observed in the current study, seem to point towards rs13447455 as a likely regulatory variant in this region. 

*UIMC1*, also known as *RAP80*, encodes a protein that plays an essential role in the DNA repair pathway via its indirect interaction with BRCA1 [36]. Four haplotypes were estimated for the *UIMC1* promoter region. As shown in Figure 2B, transient transfection showed a significant difference in transcriptional activity between the H1 and H3 haplotypes, which differ at the V7-rs7726380. EMSA showed a clear-cut differential binding of nuclear proteins to the probe carrying the C allele of rs7726380 in MCF7 cells (Figure 4). TFBS prediction analysis suggested that rs7726380 may potentially alter the putative binding site of two transcription factors, namely p53 and OCT1. However, supershift experiments did not provide any evidence of binding between antibodies to either of the two candidate transcription factors and rs7726380. In HeLa cells, a significant, albeit more modest, decrease in the expression level of the H3 rHap was also observed. However, no clear difference in the EMSA pattern of binding observed with the probe carrying the C allele could account for the latter decrease in activity. On the other hand, EMSA analysis indicates that an as yet unknown transcription factor binds to the wild-type allele. Thus, the mechanism involved in the regulation of *UIMC1* expression appears to differ between the two cell lines and could be modulated by the interaction with additional transcription factors that remain to be identified. 

We also analyzed the promoter region of the *ATR*, *PALB2*, *RAD51C* and *MRE11A* genes. We observed a significant modulation of promoter transcriptional activity for these four genes in both cell lines tested (Figure S1). The H2 ATR haplotype differs significantly from H1 at V6; the H6 and H8 *PALB2* haplotypes are different from H1 at V5, V7, V8, V10, and V11; and the H2, H3 and H4 *RAD51C* haplotypes differ from H1 at V1 and V2. Finally, for *MRE11A*, the expression level of H7 was significantly different from that seen for the wild-type H1, which might be due to V1 and V2. For these four genes, the haplotypes associated with altered gene expression level differed from the reference haplotype (H1) by multiple SNPs, which made it impractical to infer which, if any, of the individual SNPs was primarily responsible for the observed differences in transcriptional activity and prevented us from performing further functional studies using the type of approach used for the other gene promoters described above.

## 4. Discussion

More than 90% of the common genetic trait-associated variations identified by genome-wide association studies lie in non-coding DNA regions [37,38]. Thus, most of the variants identified are causally associated with complex diseases through effects on gene expression levels. 

In this study, we identified several biologically plausible functional variations in the promoter region of genes encoding proteins involved in steroid action, cell cycle control and DNA damage response. Using the rigorous combination of luciferase reporter gene-assisted gene expression, in silico transcription factor prediction, EMSA and supershift assays herein described, we have shown that several variants identified for the relevant genes under study may act as regulatory variants that alter the transcriptional activity of their respective genes via either an increase or a decrease of gene expression levels. Indeed, our data indicate that haplotypes carrying the minor alleles of rs75311867, rs1271572, rs35237183, rs7726380 and rs13447455 significantly altered the respective promoter activity compared to the reference (i.e., wild-type) haplotype (H1) of the *ESR1*, *ESR2*, *FOXA1*, *UIMC1* and *CDC7* genes, respectively. We also showed that altered expression levels of the *ATR*, *PALB2*, *RAD51C* and *MRE11A* genes might result from the effect of promoter-associated SNPs. 

The most interesting observations resulting from these analyses were for the *CDC7* gene. *CDC7* encodes a widely expressed protein kinase implicated in cell division, cell cycle checkpoint mechanisms, and cancer progression. Interestingly, two studies have previously reported higher CDC7 expression in cancerous compared to normal breast epithelium [39,40]. Furthermore, functional studies of CDC7 in cancer cell lines have suggested that inactivation of CDC7 causes growth arrest and cell death preferentially in cancer cells [41] while in contrast, depletion of CDC7 in normal cells led to cell cycle arrest without inducing cell death. Interestingly, recent results from the Breast Cancer Association Consortium (BCAC) show marginal evidence of association between rs13447455 and decreased overall breast cancer risk (OR = 0.968) *p* = 9.3r × 10^r−5^ (Table S3) [11,12]. These association results are in agreement with the functional data from gene reporter assays showing a decreased transcriptional activity of CDC7-Haplotype 2 harboring the rs13447455-G allele (Table 1; Figure 2). Our results also showed differential binding of the GLIS2 transcription factor to *CDC7*-rs13447455. GLIS2 belongs to the Gli-similar (Glis) 1–3 proteins, a sub-family of Krüppel-like zinc finger proteins [42]. It acts as a negative regulator of β-catenin [43] that regulates cell adhesion and migration through interactions with cadherins, which explains the implication of β-catenin in several cancers [44]. β-catenin is also an integral component of the canonical Wnt signaling pathway [45] known to be involved in cancer development [46]. Additional studies are needed to determine the molecular basis of the interaction between GLIS2 and *CDC7* as well as the role of rs13447455 in breast cancer susceptibility. 

On another note, it has been shown that several breast cancer associated SNPs are located in FOXA1 binding sites and alter gene expression by modulating the affinity of chromatin for FOXA1 [47]. Thus, the potential functional variant identified in this study on the promoter region of *FOXA1* (rs35237183) represents a good candidate variant for breast cancer susceptibility; however further studies are needed to confirm this.

In addition to their potential associations with breast cancer risk in the general population, polymorphisms in DNA repair genes are candidates for the modification of breast cancer risk in *BRCA1* and *BRCA2* mutation carriers, in view of the known variability in the penetrance of the latter high-risk breast cancer genes [48]. In fact, multiple polypeptides involved in the DNA repair pathway are found within multiprotein complexes formed with the BRCA1 and BRCA2 proteins [49,50]. Thus, based on the principle that haplotype analysis may increase our ability to uncover associations with complex diseases such as breast cancer, Rebbeck et al. already studied the association of DNA repair gene haplotypes with the modification of breast and ovarian cancer risk among *BRCA1* mutation carriers [51]. They detected significant associations of some haplotypes of *BABAM1*, *ATM*, *CTIP (RBBP8)*, *TOPBP1*, *BRIP1*, *BRE* and *RAD50* with the modification of breast cancer risk. They also showed that *FAM175A* and *UIMC1* haplotypes might significantly modify the risk of ovarian cancer. Some of the SNPs in these genes, such as *ATM* rs228589 and *BABAM1* rs2278256, were analyzed in the present study. We could not substantiate the observations previously made by Rebbeck et al. [51] with a functional effect of these SNPs on the level of promoter activity of the respective genes. However, the methodological design of our study was not intended to systematically analyze specific SNPs but rather to assess the mechanistic basis for the functional effect of haplotype variants of genes involved in steroid action, cell cycle regulation and the DNA damage response, by testing relatively straightforward predictions on the potential outcome of sequence mutations on transcription factor binding, and hence, promoter activity.

It is noteworthy that among the large number of polymorphisms tested in this study, only a few functional SNPs could be conclusively identified with supporting evidence. These genes, which encode proteins that are involved in steroid action, cell cycle control and DNA repair pathways, are highly conserved [52,53], and therefore, the likelihood of finding common functional variants with a large effect on these genes is almost null due to the major role they play in the maintenance of the genomic integrity. Nonetheless, while small variations in the expression level of these genes might functionally impact their expression, transient transfection assays might not be the appropriate method to detect such small effects. We are also aware that the assessment of the functional effect of the sex steroid receptor expression and DNA repair polymorphisms studied here might be more apparent after exposure to the relevant hormonal stimulation and to DNA-damaging agents such as ionizing radiation. 

Our study also had several limitations. Although the investigation of more distal SNPs would have been interesting, as it has been shown that distal SNPs can play an important role in the regulation of specific genes, this was beyond the scope of this study, which was not designed to provide an exhaustive analysis of the regulation of the selected genes but rather focused on proximal promoter SNPs. Moreover, this study was initiated in 2009. At the time of this study design, the information that is currently available in SNP databases and which helps guide the selection of SNPs for functional validation and association with disease, was rather limited.

In conclusion, the approach proposed in the present report provides original evidence that certain polymorphisms of genes involved in sex steroid receptor expression, cell cycle and DNA repair, most notably *ESR1* rs75311867, *ESR2* rs1271572, *FOXA1* rs35237183, *UIMC1* rs7726380, and *CDC7* rs13447455 may indeed play a functional role in transcriptional activity. Further molecular and epidemiological analysis of these as well as other polymorphisms affecting these pathways will clearly be required to provide more detailed insight on the in vivo role between the estrogen- as well as DNA repair-and cell cycle related pathways and carcinogenesis. Furthermore, this study provides valuable insight on the experimental challenges and limitations faced in the functional assessment of the effect of a given SNP or haplotype. As poorly reproducible allelic expression has been reported in several studies [54,55], our results and proposed approach are of relevance to overcome these challenges. The originality of the solution herein proposed to address these challenges consists of a methodological and statistical approach to perform successful gene reporter analysis that is amenable to direct experimental testing and is closely consistent with previously recommended standards such as those of Hoogendoorn et al. [34,35]. 

## Figures and Tables

**Table 1:** Results from gene reporter assays and transcriptional activity of the characterized promotor haplotypes.

		Gene Symbol	Haplotype Designation	Promotor Activity														
				MCF7								HELA						
				Renilla Luciferase Plasmid	Number of Experiments	Number of Clones	Activity Relative to Control	Relative Allele Activity to H1				Renilla Luciferase Plasmid	Number of Experiments	Number of Clones	Activity Relative to Control	Relative Allele Activity to H1		
							Fold Induction	Fold Induction	*p*-value						Fold Induction	Fold Induction	*p*-Value	
**PROMOTERS WITH DIFFERENTIAL HAPLOTYPE EXPRESSION IN TWO CELL LINES**																		
	**SEX STEROID HORMONE ACTION GENES**																	
		*ESR1*		pRL-null	5							pRL-null	4					
			pGL3-Basic			1	1.0	-						1	1.0	-		
			H1			1	7.7	1.000	-					1	6.3	1.000	-	
			H2			1	2.3	0.293	<2.2 × 10^−16^	**				1	3.6	0.564	<2.2 × 10^−16^	**
			H3			1	2.5	0.326	<2.2 × 10^−16^	**				1	3.7	0.579	<2.2 × 10^−16^	**
			H4			1	2.1	0.277	<2.2 × 10^−16^	**				1	3.1	0.496	<2.2 × 10^−16^	**
		*ESR2*		pRL-CMV	2							pRL-CMV	2					
			pGL3-Basic			1	1.0	-						1	1.0	-		
			H1			1	2.0	1.000	-					1	2.8	1.000	-	
			H3			1	5.0	2.544	2.9 × 10^−04^	**				1	7.3	2.582	8.3 × 10^−11^	**
			H6			1	6.0	3.053	9.0 × 10^−06^	**				1	8.4	2.967	1.5 × 10^−12^	**
		*FOXA1*		pRL-CMV	1							pRL-null	2					
			pGL3-Basic			1	1.0	-						1	1.0	-		
			H1			2	69.0	1.000	-					1	5.8	1.000	-	
			H2			2	65.0	0.942	1.0					1	4.0	0.699	3.0 × 10^−10^	**
			H3			3	63.2	0.798	5.9 × 10^−04^	**				1	3.6	0.619	3.1 × 10^−13^	**
			H4			2	64.5	0.935	1.0					1	5.4	0.938	3.8 × 10^−01^	
			H5			2	52.6	0.762	3.7 × 10^−06^	**				1	4.5	0.781	6.1 × 10^−07^	**
			H6			2	62.8	0.909	2.9 × 10^−01^					1	4.6	0.806	6.9 × 10^−06^	**
	**DNA REPAIR & CELL CYCLE CONTROL GENES**																	
		*ATR*		pRL-CMV	4							pRL-CMV	4					
			pGL3-Basic			1	1.0	-						1	1.0	-		
			H1			1	164.0	1.000	-					1	133.3	1.000	-	
			H2			1	116.9	0.729	3.7 × 10^−07^	**				1	103.4	0.775	1.2 × 10^−05^	**
		*CDC7*		pRL-null	1							pRL-null	2					
			pGL3-Basic			1	1.0	-						1	1.0	-		
			H1			3	131.5	1.000	-					3	193.2	1.000	-	
			H2			4	57.2	0.435	<2.2 × 10^−16^	**				4	146.0	0.756	<2.2 × 10^−16^	**
			H3			3	132.8	1.011	1.0					3	168.3	0.871	1.6 × 10^−12^	**
		*MRE11A*		pRL-CMV	1							pRL-null	1					
			pGL3-Basic			1	1.0	-						1	1.0	-		
			H1			5	116.7	1.000	-					3	7.8	1.000	-	
			H4			5	115.5	0.990	1.0					3	5.9	0.753	2.5 × 10^−12^	**
			H6			3	115.0	0.985	1.0						-	-		
			H7			3	132.9	1.139	4.5 × 10^−03^	*				3	8.6	1.097	9.6 × 10^−04^	**
			H8			3	115.1	0.987	1.0					3	6.6	0.844	2.3 × 10^−07^	**
		*PALB2*		pRL-CMV	2							pRL-CMV	3					
			pGL3-Basic			1	1.0	-						1	1.0	-		
			H1			3	34.1	1.000	-					1	50.0	1.000	-	
			H6			3	25.1	0.737	1.8 × 10^−12^	**				1	40.1	0.803	9.9 × 10^−06^	**
			H8			3	23.5	0.689	2.7 × 10^−15^	**				1	40.2	0.803	1.0 × 10^−05^	**
		*RAD51c*		pRL-null	1							pRL-CMV	3					
			pGL3-Basic			1	1.0	-						1	1.0	-		
			H1			2	346.0	1.000	-					1	43.7	1.000	-	
			H2			2	274.7	0.794	1.9 × 10^−08^	**				1	56.0	1.280	1.4 × 10^−06^	**
			H3			1	240.5	0.695	6.4 × 10^−10^	**				1	49.5	1.130	2.5 × 10^−02^	
			H4			2	256.4	0.741	3.1 × 10^−10^	**				1	42.7	0.976	1.0	
		*UIMC1*		pRL-null	2							pRL-CMV	2					
			pGL3-Basic			1	1.0	-						1	1.0	-		
			H1			1	38.3	1.000	-					1	25.9	1.000	-	
			H2			1	43.1	1.125	4.0 × 10^−01^					1	26.8	1.035	9.2 × 10^−01^	
			H3			1	26.5	0.693	2.3 × 10^−03^	*				1	16.9	0.651	1.9 × 10^−10^	**
			H4			1	31.7	0.828	1.3 × 10^−01^					1	23.0	0.889	8.3 × 10^−03^	*
**PROMOTERS WITH DIFFERENTIAL HAPLOTYPE EXPRESSION IN ONE CELL LINE**																		
	**SEX STEROID HORMONE ACTION GENES**																	
		*PGR*		pRL-CMV	3							pRL-CMV	2					
			pGL3-Basic			1	1.0	-						1	1.0	-		
			H1			1	2.3	1.000	-					1	1.4	1.000	-	
			H2			1	2.1	0.909	5.3 × 10^−01^					1	3.9	2.871	<2.2 × 10^−16^	**
			H6			1	2.1	0.938	1.0					1	3.9	2.803	<2.2 × 10^−16^	**
			H7			1	2.3	1.001	1.0					1	3.8	2.731	<2.2 × 10^−16^	**
			H10			1	2.3	1.009	1.0					1	4.4	3.184	<2.2 × 10^−16^	**
	**DNA REPAIR & CELL CYCLE CONTROL GENES**																	
		*BABAM1*		pRL-CMV	4							pRL-CMV	4					
			pGL3-Basic			1	1.0	-						1	1.0	-		
			H1			1	100.0	1.000	-					1	19.0	1.000	-	
			H2			1	127.0	1.271	5.6 × 10^−02^					1	17.1	0.896	4.2 × 10^−02^	
			H3			1	134.8	1.348	1.1 × 10^−02^					1	16.2	0.852	2.8 × 10^−03^	*
		*BRCC3*		pRL-CMV	2							pRL-CMV	2					
			pGL3-Basic			1	1.0	-						1	1.0	-		
			H1			1	24.8	1.000	-					1	104.3	1.000	-	
			H2			1	30.4	1.226	1.0 × 10^−04^	**				1	128.7	1.234	5.0 × 10^−02^	
			H3			1	17.7	0.715	3.3 × 10^−06^	**				1	90.5	0.867	4.8 × 10^−01^	
			H4			1	24.3	0.979	1.0					1	117.5	1.127	5.4 × 10^−01^	
		*BRE*		pRL-CMV	2							pRL-CMV	2					
			pGL3-Basic			1	1.0	-						1	1.0	-		
			H1			3	175.9	1.000	-					1	10.9	1.000	-	
			H2			2	91.8	0.522	7.8 × 10^−08^	**				1	11.0	1.011	1.0	
			H3			3	117.9	0.670	2.7 × 10^−05^	**				1	5.1	0.467	1.2 × 10^−02^	
			H4			2	64.3	0.365	4.4 × 10^−12^	**				1	6.0	0.555	4.6 × 10^−02^	
			H5			2	160.8	0.915	1.0					1	3.4	0.311	8.9 × 10^−04^	**
		*NELFB*		pRL-CMV	2							pRL-CMV	2					
			pGL3-Basic			1	1.0	-						1	1.0	-		
			H1			1	1047.8	1.000	-					1	205.7	1.000	-	
			H7			1	982.7	0.938	3.0 × 10^−01^					1	222.1	1.080	5.6 × 10^−03^	*
		*NBN*		pRL-CMV	3							pRL-null	1					
			pGL3-Basic			1	1.0	-						1	1.0	-		
			H1			3	68.5	1.000	-					3	8.5	1.000	-	
			H2			3	75.3	1.099	2.6 × 10^−02^					3	13.4	1.577	<2.2 × 10^−16^	**
			H3			3	67.1	0.979	1.0					3	14.7	1.729	<2.2 × 10^−16^	**
			H4			3	41.3	0.603	<2.2 × 10^−16^	**				3	9.1	1.069	9.5 × 10^−02^	
		*RAD51*		pRL-CMV	1							pRL-CMV	2					
			pGL3-Basic			1	1.0	-						1	1.0	-		
			H1			3	235.4	1.000	-					1	163.5	1.000	-	
			H5			2	259.8	1.103	3.0 × 10^−03^	*				1	162.1	0.992	1.0	
			H6			3	237.2	1.007	1.0					1	137.2	0.839	1.3 × 10^−03^	*
		*RNF8*		pRL-CMV	2													
			pGL3-Basic			1	1.0	-				pRL-CMV	2		1.0	-		
			H1			1	7.4	1.000	-					1	3.1	1.000	-	
			H3			1	8.0	1.081	9.5 × 10^−01^					1	3.2	1.035	1.0	
			H5			1	8.2	1.116	3.9 × 10^−01^					1	3.1	1.000	1.0	
			H6			1	5.8	0.792	1.7 × 10^−02^					1	3.1	1.019	1.0	
			H10			1	5.5	0.744	2.5 × 10^−03^	*				1	3.0	0.987	1.0	
		*RPS6KA2*		pRL-null	3							pRL-null	2					
			pGL3-Basic			1	1.0	-						1	1.0	-		
			H1			6	9.3	1.000	-					6	9.0	1.000	-	
			H2			5	8.5	0.920	1.0					5	8.2	0.908	3.9 × 10^−05^	**
			H3			6	10.0	1.088	9.7 × 10^−01^					6	10.8	1.194	<2.2 × 10^−16^	**
**PROMOTERS WITH NO DIFFERENTIAL HAPLOTYPE EXPRESSION**																		
	**DNA REPAIR & CELL CYCLE CONTROL GENES**																	
		*ATM*		pRL-CMV	3							pRL-CMV	3					
			pGL3-Basic			1	1.0	-						1	1.0	-		
			H1			1	366.3	1.000	-					1	295.3	1.000	-	
			H2			1	375.8	1.026	1.0					1	328.3	1.112	3.8 × 10^−02^	
			H3			1	346.9	0.947	5.9 × 10^−01^					1	309.4	1.048	6.0 × 10^−01^	
		*BRIP1*		pRL-null	1							pRL-CMV	1					
			pGL3-Basic			1	1.0	-						1	1.0	-		
			H1			3	68.8	1.000	-					3	7.3	1.000	-	
			H2			3	69.9	1.016	1.0					3	6.9	1.065	1.1 × 10^−02^	
			H4			3	63.7	0.926	3.6 × 10^−01^					3	6.9	0.944	2.5 × 10^−02^	
		*H2AFX*		pRL-CMV	2							pRL-CMV	2					
			pGL3-Basic			1	1.0	-						1	1.0	-		
			H1			1	273.9	1.000	-					1	250.4	1.000	-	
			H2			1	276.8	1.011	1.0					1	242.0	0.966	5.8 × 10^−01^	
			H3			1	302.1	1.103	3.0 × 10^−02^					1	271.9	1.086	2.3 × 10^−02^	
		*UBE2N*		pRL-CMV	2							pRL-CMV	2					
			pGL3-Basic			1	1.0	-						1	1.0	-		
			H1			1	2.0	1.000	-					1	3.4	1.000	-	
			H4			1	2.1	1.076	3.6 × 10^−01^					1	3.3	0.763	1.0	
			H5			1	2.0	1.019	1.0					1	3.7	0.875	3.2 × 10^−01^	
			H6			1	1.9	0.925	3.7 × 10^−01^					1	3.3	0.771	1.0	
																		
																	* p < 0.01	
																	** p < 0.001	
																		

**Table 2:** Altered transcription factor binding sites and corresponding antibodies tested in Electrophoretic Mobility Shift Assays.

	Gene Symbol	Variant #	Observed Variation	Rs ID *		EMSA Analysis						
						Altered TFBS (MatInspector)			Gel Shift		Super Shift	
						Effect	Name of TFBS		Observed Binding		Antibody (Santa Cruz)	Observed Protein Binding
**SEX STEROID HORMONE ACTION GENES**												
	***ESR1***											
		v1	insAA	rs75311867		GAIN	Myelin Transcription factor		YES		Myt1 (N-13)	NO
	***ESR2***											
		v4	T>G	rs1271572		LOOSE	Poz (BTB) and AT hook containing zinc finger		YES		PaTZ1 (G-13)	NO
		v5	insG	rs66615803		LOOSE	Zebrafish PAX9 binding sites		NO			
						LOOSE	GLIS family zinc finger 3, Gli-similar 3		NO			
						GAIN	MYC-associated zinc finger protein related transcription factor		NO			
						GAIN	Zinc finger transcription factor, Zic family member 2 (odd-paired homolog, Drosophila)		NO			
	***FOXA1***											
		v5	delAGA	rs35237183		GAIN	Forkhead domain factors		YES		HNF3-alpha	NO
		v6	C>A	rs10145379		GAIN	GC-Box factors SP1/GC		NO			
						LOOSE	DM domain-containing transcription factors		NO			
						LOOSE	Bromodomain and PHD domain transcription		NO			
**DNA REPAIR & CELL CYCLE CONTROL GENES**												
	***CDC7***											
		v7	C>T	rs13447450		NO CHANGE						
		v10	A>G	rs13447455		GAIN	Paired box factor 5		YES		PAX5 (C-20)	NO
						GAIN	Wilms tumor 1		YES		WT1 (C-19)	NO
						GAIN	GLi-similar zinc finger		YES		GLIS2 (E-17)	YES
						LOOSE	Myeloid zinc finger		NO			
	***UIMC1***											
		v7	T>G	rs7726380		GAIN	p53 tumor suppressors		YES		P53 (Pab1801)X	NO
						LOOSE	POU Class 2 homeobox 1		YES		OCT-1 (C-21)	NO
												

* dbSNP build 138.

## Supplementary Materials

The following are available online at https://www.mdpi.com/2073-4425/10/3/186/s1, Figure S1. Assessment of the functional impact of *ATR*, *PALB2*, and *RAD51C* and *MRE11A* gene promoter variants on promoter activity using gene reporter assays. Statistically significant differences were detected between haplotypes which exhibit relatively large standard errors, especially for the *RAD51C* gene. This stresses the importance of distinctly modelling the variability due to the experiment number and haplotype by means of the mixed model, Table S1. Wild-type and variants haplotypes within the promotor region of genes involved in sex steroid action, DNA repair and cell cycle control; Table S2. Sequence of PCR primers designed for the amplification of promotor region of the 24 candidate genes, Table S3. Associations of promoter SNPs with overall breast cancer risk. Associations are shown for SNPs from promoters showing differential haplotype expression in at least one cell line. Data was obtained from the Breast Cancer Association Consortium (*Michailidou K.* et al. *Nat Genet. 2013 45(4):353-61; Michailidou K.* et al. *Nature. 2017 551(7678):92-94*). **Supplementary Methods.** The statistical analyses have been mainly performed using the Statistical Analysis System (SAS). Here we show an example of a SAS code that have been used in this study when analyzing the PALB2 data (Figure 1): proc mixed data = PALB2; class expNo cloneHap; model Activity = cloneHap/s ddfm = kr outpm = stat vciry; random expNo/G s; lsmeans cloneHap/e; lsmestimate cloneHap ’H1(3) vs. H1(2)’ -1 1 0 0 0 0 0 0 0, ’H1(4) vs. H1(2)’ -1 0 1 0 0 0 0 0 0, ’H6(12) vs. H6(11)’ 0 0 0 -1 1 0 0 0 0, ’H6(13) vs. H6(11)’ 0 0 0 -1 0 1 0 0 0, ’H8(17) vs. H8(16)’ 0 0 0 0 0 0 -1. 1 0, ’H8(18) vs. H8(16)’ 0 0 0 0 0 0 -1 0 1, ’H6 vs. H1’ -0.33 -0.33 -0.33 0.33 0.33 0.33 0 0 0, ’H8 vs. H1’ -0.33 -0.33 -0.33 0 0 0 0.33 0.33 0.33 / adjust = bon; run.
