# Supplementary material for: Functional Analysis of Promoter Variants in Genes Involved in Sex Steroid Action, DNA Repair and Cell Cycle Control
Source: Genes (Basel). 2019 Feb 28;10(3):186. doi: 10.3390/genes10030186 (PMC6470759; doi:10.3390/genes10030186)
Supplement: Supplementary file 1 [file genes-10-00186-s001.zip › genes-421015-supplementary-figure-1.doc]

**Figure S1.** Assessment of the functional impact of *ATR, PALB2*, and *RAD51C* and *MRE11A* genes promoter variants on promoter activity using gene reporter assays.
